# How Female Mice Attract Males: A Urinary Volatile Amine Activates a Trace Amine-Associated Receptor That Induces Male Sexual Interest

**DOI:** 10.3389/fphar.2018.00924

**Published:** 2018-08-15

**Authors:** Anja Harmeier, Claas A. Meyer, Andreas Staempfli, Fabio Casagrande, Marija M. Petrinovic, Yan-Ping Zhang, Basil Künnecke, Antonio Iglesias, Oliver P. Höner, Marius C. Hoener

**Affiliations:** ^1^Roche Pharma Research and Early Development, Roche Innovation Center Basel, F. Hoffmann-La Roche Ltd., Basel, Switzerland; ^2^Department of Forensic and Neurodevelopmental Sciences, Sackler Institute for Translational Neurodevelopment, Institute of Psychiatry, Psychology and Neuroscience, King’s College London, London, United Kingdom; ^3^Department of Evolutionary Ecology, Leibniz Institute for Zoo and Wildlife Research, Berlin, Germany; ^4^Department of Neurosymptomatic Domains, Neuroscience, Ophthalmology and Rare Diseases Discovery and Translational Area, Roche Pharma Research and Early Development pRED, Roche Innovation Center Basel, F. Hoffmann-La Roche Ltd., Basel, Switzerland

**Keywords:** trace amine-associated receptor, TAAR3, volatile amine, isobutylamine, odor recognition, mate choice, sexual interest

## Abstract

Individuals of many species rely on odors to communicate, find breeding partners, locate resources and sense dangers. In vertebrates, odorants are detected by chemosensory receptors of the olfactory system. One class of these receptors, the trace amine-associated receptors (TAARs), was recently suggested to mediate male sexual interest and mate choice. Here we tested this hypothesis in mice by generating a cluster deletion mouse (*Taar2-9*^−/−^) lacking all TAARs expressed in the olfactory epithelium, and evaluating transduction pathways from odorants to TAARs, neural activity and behaviors reflecting sexual interest. We found that a urinary volatile amine, isobutylamine (IBA), was a potent ligand for TAAR3 (but not TAAR1, 4, 5, and 6). When males were exposed to IBA, brain regions associated with sexual behaviors were less active in *Taar2-9*^−/−^ than in wild type males. Accordingly, *Taar2-9*^−/−^ males spent less time sniffing both the urine of females and pure IBA than wild type males. This is the first demonstration of a comprehensive transduction pathway linking odorants to TAARs and male sexual interest. Interestingly, the concentration of IBA in female urine varied across the estrus cycle with a peak during estrus. This variation in IBA concentration may represent a simple olfactory cue for males to recognize receptive females. Our results are consistent with the hypothesis that IBA and TAARs play an important role in the recognition of breeding partners and mate choice.

## Introduction

Survival and reproductive success of individuals strongly depend on their ability to locate resources, sense dangers, and find breeding partners. In many species, odors are key cues for the detection of resources and dangers such as pathogens, predators, and toxins (Ferrero et al., 2011; Li and Liberles, 2015). Increasing evidence from studies on mammals (including humans) suggests that odors also play an important role in the recognition and attraction of breeding partners and mate choice (Baum and Kelliher, 2009; Milinski et al., 2013). However, we currently know little about the odorants and respective receptors that detect the quality and reproductive state of breeding partners and the transduction pathway that links odorants to behaviors associated with mate choice (Li and Liberles, 2015).

In mammals, odorants are detected by ORs and vomeronasal receptors expressed in three structures of the olfactory system: the MOE, the VNO, and the Grueneberg ganglion (Mombaerts, 2001; Stowers and Logan, 2010). Vomeronasal receptors type 1 and 2 as well as formyl peptide receptors expressed by VNO neurons project to the accessory olfactory bulb and have been shown to elicit sexual behavior and aggression in mice when activated (Del Punta et al., 2002; Liberles, 2014). ORs expressed by Grueneberg ganglion neurons detect alarm pheromones and induce freezing behavior in mice (Brechbuhl et al., 2008). ORs are expressed by neurons of the MOE. These neurons project their axons to the olfactory bulb where they form glomeruli in discrete domains. The ORs are divided into three classes: class I OR, class II OR, and TAARs (Kobayakawa et al., 2007; Bozza et al., 2009; de Castro, 2009; Pacifico et al., 2012; Gainetdinov et al., 2018). The mammalian TAAR G-protein coupled receptor family consists of 9 main members, of which all but TAAR2 have the coding sequences located on a single exon on chromosome 10 (Lindemann et al., 2005; Gainetdinov et al., 2018). All TAARs detect primary, secondary and/or tertiary amines, while only TAAR1 and TAAR4 bind trace amines (e.g., β-PEA or *p*-tyramine) (Lindemann et al., 2005; Liberles and Buck, 2006; Ferrero et al., 2012; Gainetdinov et al., 2018). TAAR1 is expressed in a few, distinct regions of the brain, the β-cells of the pancreas, the stomach, and the intestine (Revel et al., 2013). It modulates the dopaminergic and serotonergic systems in response to endogenous trace amine levels (Lindemann et al., 2008; Revel et al., 2011; Berry et al., 2017), regulates processes associated with reward, cognition and mood (Revel et al., 2013). It also mediates incretin-like effects resulting in glucose control, improved insulin sensitivity, reduced food intake and decreased body weight (Raab et al., 2016; Berry et al., 2017).

The eight main TAARs that are expressed by MOE neurons (TAARs 2 to 9) (Liberles and Buck, 2006; Carnicelli et al., 2010; Pacifico et al., 2012; Horowitz et al., 2014; Kanageswaran et al., 2015) detect odorants of diverse ecological and ethological origin (Ferrero et al., 2011; Dewan et al., 2013; Li et al., 2013; Liberles, 2015; Gainetdinov et al., 2018). TAAR4 is activated by the trace amine β-PEA, a kairomone identified in the urine of most carnivores, and induces an aversive behavioral response in mice (Ferrero et al., 2011). TAAR5 is stimulated by TMA, an amine present at high concentrations in the urine of adult male mice and in its oxidized form in the urine of females (Li et al., 2013). TMA-activated TAAR5 enables males to recognize conspecifics and their sex and age (Li et al., 2013). Interestingly, a recent study on bats found TAAR3 to mediate major histocompatibility complex-dependent mate choice (Santos et al., 2016).

Here we tested the hypothesis that TAARs are involved in mate choice by examining odorants and receptors that indicate and detect the reproductive state of breeding partners and by evaluating transduction pathways that link odorants to sexual behaviors associated with mate choice. We generated a TAAR cluster deletion (*Taar2-9*^−/−^) mouse line lacking all TAARs expressed in the olfactory epithelium and tested which odorants in the urine of mice are sex-specific, whether their concentration varies during the estrus cycle; and which TAARs detect these odorants. To determine whether TAAR activation in the olfactory epithelium translates into changes of neural activity, and to gain insights on the projection areas of TAAR-expressing olfactory neurons, we exposed *Taar2-9*^−/−^ males and their WT littermates to odors and compared their brain activity patterns. We then measured behaviors associated with sexual interest. Previous studies showed that the time male mice spend sniffing an odor reflects their interest in that odor (Muroi et al., 2006; Malkesman et al., 2010). When a male mouse recognizes the odor of a female mouse in estrus, it starts vocalizing in the ultrasonic range. These courtship calls attract mating partners both in laboratory (Whitney and Nyby, 1979) and wild (Musolf et al., 2010) mice. We therefore also compared both the sniffing behavior and the type and quality of courtship vocalizations of *Taar2-9*^−/−^ and WT males when they were exposed to specific urinary components or whole urine of female mice.

## Materials and Methods

### Animals

All animal experiments were performed at F. Hoffmann-La Roche Ltd. (Basel, Switzerland) in compliance with the Swiss federal regulations on animal protection and the rules of the Association for Assessment and Accreditation of Laboratory Animal Care International (AAALAC), and with the explicit approval by the Basel Cantonal veterinary authorities. The mice were maintained in an enriched environment with a 12 h light/12 h dark cycle (lights on from 6:00 am to 6:00 pm) and temperature (2–24 °C) and humidity (50–60%) controlled conditions; food and water were available *ad libitum*. The mice were handled to a minimum and singly housed 1 week before and for the duration of the experiments. Behavioral experiments were performed with adult male mice at the age of 12 weeks. For social cues, age-matched male and female mice were used as “baits.” Prior to social cue experiments the stage of the estrus cycle of each female was determined to ensure that the odor of mice in estrus were included in the experiments.

### Gene Targeting

**FIGURE 1:**
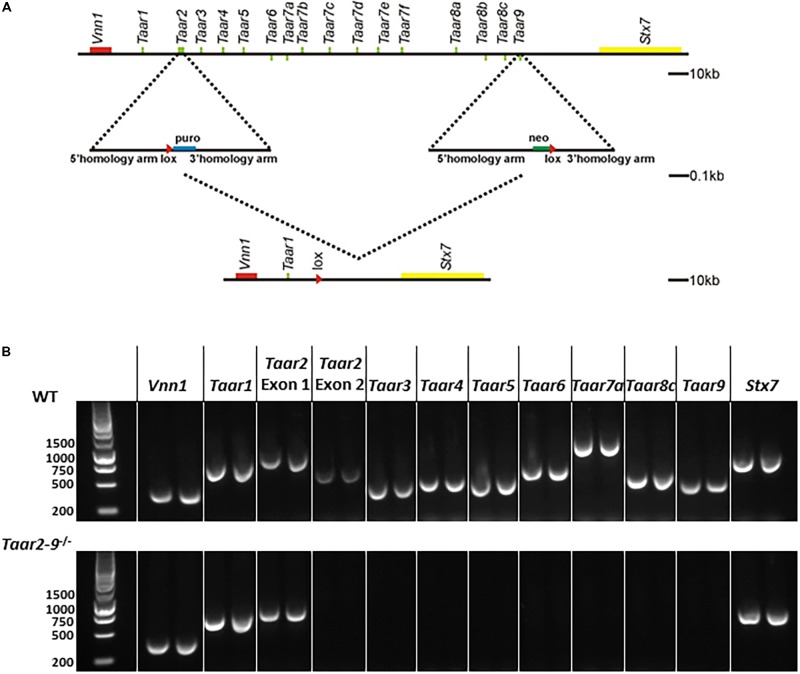
Generation of *Taar2-9* cluster deletion mice. **(A)** Overview of the chromosomal deletion method. Similar to human and rat, the entire family of mouse TAAR genes maps to a narrow region of a single chromosome (Li et al., 2013). The murine *Taar* cluster (Lindemann et al., 2005) and neighboring genes (Vnn1, Stx7) are shown. A recognition sequence (loxP, red triangle) for the Cre site specific recombinase was inserted into the *Taar2* gene using a targeting vector with homology arms flanking a puromycin selection (puro) and homologous recombination. Similarly, a second loxP sequence was inserted into the *Taar9* gene using neomycin (Neo) selection. Cre recombination was used to delete 170 kb of the murine chromosome 10. This includes all *Taar* genes except *Taar1* and *Taar2* Exon1. There are no known other genes or RNAs encoded in this region. **(B)** PCRs from genomic DNA from tail biopsies using oligonucleotides encompassing the *Taar* genes *Taar1, Taar2* Exon1 and Exon2, *Taar3, Taar4, Taar5, Taar6, Taar7a, Taar8c, Taar9* and the flanking *Vnn1* and *Stx7* produced PCR fragments of the correct size in the wild type (WT) samples, but not for *Taar2* Exon2 until *Taar9* in the *Taar2-9*^−/−^ samples.

The cluster deletion (*Taar2-9*^−/−^) mouse line was engineered by homologous recombination using methods described previously (Lindemann et al., 2008) with modifications. In mouse as in rat and human, all *Taar*-encoding genes except for *Taar2* have their coding sequences located on a single exon with similar length (Lindemann and Hoener, 2005). Based on this, 2 targeting vectors with loxP sites were generated for genomic fragments located 5′ and 3′ from *Taar2* Exon2 or *Taar9* coding sequence including puromycin or neomycin resistance, respectively (see **Figure 1A**). The linearized vectors were electroporated into C57BL/6 embryonic stem cells and antibiotic resistant embryonic stem cells were selected, identified by PCR and used for the generation of chimeras according to standard protocol (Joyner, 1999). Only the crossing of the chimeras with a mother having a cre-positive germline resulted in the successful generation of *Taar2-9*^−/−^ mice. The recombinant alleles were maintained in a pure C57BL/6 background. Heterozygous mice were bred for the generation of homozygous *Taar2-9*^−/−^ and WT littermates for behavioral tests.

The correct homologous recombination and deletion of all *Taars* but *Taar1* was further confirmed by PCR amplification of genomic DNA extracted from tail biopsies of *Taar2-9*^−/−^ mice. The following primers were used: *Vnn1*, fw: GCA GTC TTT GGA CTT CAG CAT G, rev: GCT AAT GTA GTG AGC GGA ACT G; *Taar1*, fw: ATA ATC CTG GCC ACT CTG GTT G, rev: CAC CAT GAT CCC TAA GGT CTT C; *Taar2 (Exon1)*, fw: TTT GAA GCC CAG CAG GTA CAT G, rev: CCC TAC CTT GGG ATA CTT TCA C; *Taar2 (Exon2)*, fw: CAC CCA TCA CTA TCC CTA AAG, rev: GCC ATA TAG TAT GGT CAG GTC; *Taar3*, fw: TGA CGT CCA TAT TCC ACC TGT G, rev: TTT GTG TTC GCA GGC ATA TCG C; *Taar4*, fw: CTA CCA CAG ACT TCC TGT TGA G, rev: AAA TCC CTA CCA TGA CTG TCC C; *Taar5*, fw: AGA CAT GTT GCT AGG TCT GCT G; rev: AGC TGA TCA TGA TTA GGC AGG G, *Taar6*, fw: TCA ATG CTG TAG GGC AAC CAT G, rev: ATC GAG AGC TGC TGG TAC TTT G; *Taar7a*, fw: GCC AGT GAC AAT GAG CTT GAT G, rev: CTG TTC TCT GCC ACA TCT ACA G*; Taar8c*, fw: TCA CCT CTG TTG CCA CTT ACA G, rev: TTC AGC ATG GTC AGG TCC ATT G*; Taar9*, fw: GCT GTA CCC TCT ATC TTC CTA G, rev: AGT TCC ACA CGT GTT TCG ACA C; *Stx7*, fw: CCA GGT GAT ATT TCG TGT CCA G, rev: CAA CGC ATC TCC TCT AAC ATC C.

### Recognition of Odors

To investigate whether naïve *Taar2-9*^−/−^ and WT male mice differed in their sensitivity to mouse odors, synthetic odors (almond, banana, TMA, β-PEA), and a control odor (water) we performed olfaction habituation/dishabituation tests adapted from Ricceri et al. (2007), Yang and Crawley (2009), and Malkesman et al. (2010). Tests were carried out in rooms with light intensity adjusted to 150 Lux after pre-habituating the mice to the test cages during a period of 30 min. Mouse odors were obtained by swiping an applicator 3 times over the bottom of cages that were inhabited by 3 or 4 non-familiar male or female mice of the same genetic background during 5 days. On the test date the estrus cycle stage of female mice was determined to insure that the cage consisted of at least one female in estrus on the test date, while other mice were potentially in estrus on the days before. Synthetic and control odors were obtained by soaking a cotton-tipped wooden applicator in 100 μL benzaldehyde (almond odor), isoamyl acetate (banana imitation), TMA (5 mM), β-PEA (5%) and distilled water. Each odor was freshly prepared and presented over the duration of 2 min. The presentation was repeated 1 or 2 times with 1 min intervals (hence, 2 or 3 presentations in total) to test habituation and dishabituation of the mice. The time spent sniffing the cotton part of the applicator during each 2 min trial was measured using a stopwatch.

To establish which amines induce differences in behavior associated with sexual interest, *Taar2-9*^−/−^ and WT male mice were exposed to odors containing two mixtures of three amines each in a range of concentrations that reflect the range of concentrations found in the urine of the study animals. In a second step, the mice were exposed to odors containing single amines of the mixture of amines that induced a difference in behavior.

The odor recognition experiments were done with male mice that had no prior sexual experience to prevent any potential influence of sexual experience on odor recognition and olfactory learning (Swaney et al., 2007).

### Nuclear Magnetic Resonance (NMR) and Gas Chromatography Mass Spectrometry (GC-MS)

To identify sex-specific volatiles and assess differences in the concentration of volatiles between the sexes and across the estrus cycle of the female, we collected urine samples from age-matched male and female WT mice. Urine of females was collected at different stages of the estrus cycle. For the discrimination of the estrus cycle stage, a microscopic analysis of the vaginal secretion was performed as described by Caligioni (2009). We focused on urinary odorants because urine is known to be a rich source of social cues (Liberles, 2009). Furthermore, intensive sniffing of the urine of females by males (“flehmen”) was observed in many taxa including carnivores, primates, marsupials and ungulates and has been related to chemosensory detection of the estrus of females (Hart, 1983).

40 μl of freshly collected urine of C57BL/6 mice were mixed with 100 μl of phosphate buffer solution (0.1 M Na_2_HPO_4_/0.02 M NaH_2_PO_4_, pH = 7.4, 0.1% NaN_3_, 2 mM 3-trimethylsilyl-1-[2,2,3,3,-2H_4_). Ten microliters of D_2_O were added to provide the field frequency deuterium lock and the total of 150 μl were transferred into 3 mm disposable NMR tubes. For the identification of amines in urine spectra, a series of selected amine references were produced at final concentrations of 5 mM under the same conditions. All urine samples were submitted for GC-MS analysis immediately after NMR measurements. All NMR spectra were recorded on a Bruker 600 MHz Avance II spectrometer equipped with a cryogenic QCI probe head at a temperature of 300 K. Spectrometer operation and data processing was done by use of Topsin 2.1 (Bruker, Fällanden). In all spectra the water signal was suppressed by a 50 Hz presaturation employed during the interscan relaxation delay. This relaxation delay was set to 2 s and 1.5 s for 1D and 2D spectra, respectively. 1D ^1^H spectra were acquired for all samples with 64 scans, a sweep width of 20 ppm, and acquisition time of 1.3 s. The quantification of the creatinine singlet at 4.06 ppm was used for the normalization of urine metabolite concentrations of NMR and GC-MS experiments.

For the GC-MS analysis, the 100 μl phosphate buffered saline including 5 μl mouse urine was directly derivatized according to methods described previously (Fernandes et al., 2001). Full scan and later single ion monitoring (SIM) GC-MS were recorded on an Agilent MSD 5973i using a DB-624 (30 m × 0.25 mm × 1.4 μm).

### cAMP Detection Assays

Measurement of cAMP concentrations were performed as described previously (Revel et al., 2011). In brief, HEK293 cells stably expressing mouse TAAR1, TAAR4, TAAR5, TAAR6 or transiently TAAR3 and Gαolf were generated, plated on 96-well plates (BIOCOAT 6640; Becton Dickinson), and incubated for 20 h at 37°C. Prior to the stimulation of the cells with a broad concentration range of specific agonists for 30 min at 37°C, cells were washed with PBS and preincubated with PBS containing 1 mM 3-isobutyl-1-methylxanthine (IBMX) for 10 min at 37°C and 5% CO_2_. Stimulation with 0.2% DMSO was set as basal level while a 30 μM β-PEA solution was set as maximal response. Subsequently, cells were lysed and cAMP assays were performed according to manufacturer’s instructions (cAMP kit; Upstate/Millipore). Finally, the plates were read with a luminometer (1420 Multilabel counter; PerkinElmer, Schwerzenbach, Switzerland) and cAMP concentrations calculated.

### Functional Magnetic Resonance Imaging (fMRI)

Adult male mice (*N* = 7–12 per group) were assessed either odor naïve or after exposure to either IBA or TMT for 20 min prior to fMRI. IBA (100 μl; 5 mM in water) or TMT (25 μl; undiluted) was presented on a filter paper in a Petri dish placed in a corner of a bedding-furnished type-3 cage serving as test arena. In preparation for fMRI assessments, individual animals were then placed in a restrainer containing a filter paper soaked with IBA (10 μl; 5 mM) or TMT (2 μl; undiluted), anesthesia was induced with isoflurane and subsequently maintained with a continuous intravenous infusion of etomidate as described previously (Petrinovic et al., 2016). Body temperature was maintained at 37°C via a feedback-regulated electric heating blanket. Breathing rate and concentrations of exhaled oxygen and CO_2_ were monitored continuously.

*In vivo* magnetic resonance imaging was carried out on a Biospec 9.4T/20 cm animal scanner (Bruker, Ettlingen, Germany) equipped with a whole-body resonator and a head coil. In brief, anatomical and perfusion-based (continuous arterial spin labeling) functional images were acquired, processed and analyzed according to the procedures described earlier (Kessler et al., 2012; Revel et al., 2013; Petrinovic et al., 2016). Quantitative estimates of regional cerebral perfusion were taken as a proxy of neural activity. Perfusion maps of each individual were normalized slice-wise to the brain–mean value in order to derive region-specific values independent of inter-individual differences of the animals’ global hemodynamic status, and to account for possible systemic effects affecting global perfusion values. Means of normalized perfusion in 41 bilateral regions were tested region-wise for significant differences using two-way ANOVAs with the factors genotype and odor, followed by *post hoc* contrasts for genotype differences and genotype–odor interactions.

### Ultrasonic Vocalization (USV)

The vocal behavior of adult male *Taar2-9*^−/−^ mice was assessed by examining USVs upon presentation of either water or urine of females in estrus based on already published protocols (Scattoni et al., 2008; Scattoni et al., 2011; Wohr et al., 2011; Nakagawa et al., 2012). Animals were habituated to the test apparatus, a quadratic polycarbonate box lined with Whatmanpaper. As a negative control for vocalization a water droplet was positioned in one quadrant of the Whatmanpaper and behavior and vocalization recorded for 5 min. Urine of females in estrous was freshly collected and placed on the opposing quadrant of the Whatmanpaper. USV was recorded with Avisoft software (Avisoft Bioacoustics, Glienicke, Germany) by a condenser microphone placed in one corner of the test apparatus connected to an ultrasound recording interface (Avisoft Bioacoustics, Glienicke, Germany) which was plugged to a computer. Stored waveforms were processed with custom written R scripts. The automatic detection and classification algorithm is based on the work of Holy and Guo (2005). Briefly, syllables are mainly identified by spectral purity, which is defined as the fraction of total power concentrated into a single frequency bin and exceeded 0.15. Candidate syllables separated by less than 10 ms were merged. Classification was based on characteristic temporal changes of pitch which is the dominant frequency as a function of time and revealed eight syllable types. Extremely short calls or noise were excluded from the analysis. We conducted a zero-inflated generalized linear mixed-effects model [package glmmTMB v0.1.1 (Magnusson et al., 2017)] to examine the influence of male type on the distribution of the number of calls across call types. We used the number of calls as the dependent variable, the type of male and type of call as fixed effects variables, and the identity of the male as random effects variable.

### Direct Social Interaction

To explore whether the social behavior of *Taar2-9*^−/−^ mice differs from that of WT mice, a direct social interaction test was carried out as previously described (Defensor et al., 2011; Scattoni et al., 2011). Briefly, the week prior to the direct social interaction test, male mice underwent the three chamber test. Therefore, habituation to the arena was not required. On the day of the experiment, two non-familiar males of the same genotype were placed in the arena for 10 min. Their nose–nose, nose–center, and nose–tail interactions were recorded using EthoVision software and reanalyzed manually.

### Active Place Avoidance – Spatial Cognition Test

The active avoidance task is a fear-motivated associative avoidance test based on electric current as a source of punishment. To avoid the aversive event, the mouse has to learn to predict the occurrence of an aversive event based on the presentation of a specific stimulus by actively moving to a different compartment. The measures recorded, number of avoidances (the mouse crossing to the other compartment during the stimulus signal), number of non-responses (the mouse failing to cross to the other compartment during the trial), and response latency (latency to avoid or escape), serve as an index of learning and allows memory to be assessed. The active avoidance task provides a simple way to assess associative learning and memory by testing the ability of the mouse to avoid an aversive event by learning to perform a specific behavior in response to a stimulus cue.

### Locomotor Activity

Previous data showed that TAAR1 has an impact on rodent sensitivity toward AMPH due to modulation of dopaminergic neurotransmission. *Taar1* knock-out mice display an elevated sensitivity to AMPH while TAAR1 overexpressing mice were insensitive to AMPH (Lindemann et al., 2008; Revel et al., 2012). AMPH-induced locomotor activity tests were performed as previously described (Lindemann et al., 2008). Briefly, *Taar2-9*^−/−^ mice and their WT littermates were habituated for 20 min to the open field arenas before application of d-AMPH (2.5 mg/kg i.p.) or vehicle. After placing the animals back to the same arena, the locomotor response was monitored and evaluated as the number of horizontal beam breaks (horizontal activity) over 90 min.

### Statistical Analysis

Statistical tests were performed using R software v.3.4.1 and associated packages (R Core Team, 2014), and JMP 10 (SAS Institute Inc.). Results are quoted as means ± standard error means (SEM), and probabilities are for two-tailed tests. The threshold for significance was set to α = 0.05.

## Results

### Mouse Line With Deletion of TAAR2 to TAAR9 (*Taar2-9*^-/-^)

In the *Taar2-9*^−/−^ cluster deletion mouse line an approximately 170 kb fragment of the murine chromosome 10 encompassing the coding sequences of *Taar2* to *Taar9* was deleted (**Figure 1A**). Homologous recombination in embryonic stem cells and loss of single targeted *Taars* was confirmed by PCR amplification of genomic DNA (**Figure 1B**). The deletion covered *Taar2* exon 2 to *Taar9*, while the surrounding genes *Vnn1* and *Stx7, Taar1* and the non-coding exon 1 of *Taar2* remained intact. In general, no differences were observed between *Taar2-9*^−/−^ and WT mice with regard to fertility, litter size, distribution of genotype, and sex-ratio of pups.

### Recognition of Female Odor by *Taar2-9*^-/-^ and WT Males

**FIGURE 2:**
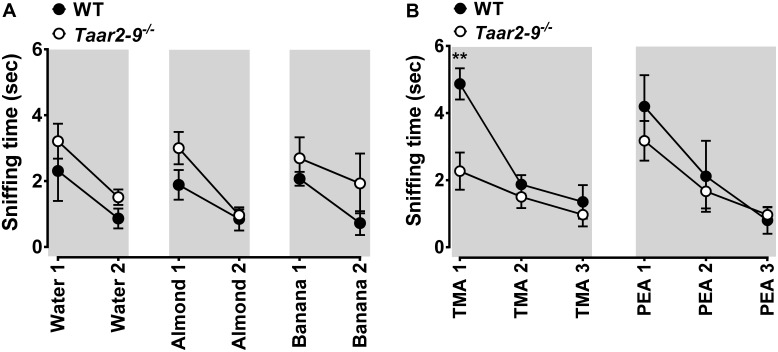
Behavioral response of wild type (WT) and *Taar2-9*^−/−^ mice to non-mouse odors. Mean (±SEM) period of time *Taar2-9*^−/−^ males (open circles) and their wild type (WT) littermates (filled circles) spent sniffing cotton-tipped wooden applicators soaked with **(A)** 100 μl distilled water, almond extract, banana imitation extract and **(B)** the volatile amines trimethylamine (TMA, 5 mM) and β-phenylethylamine (PEA, 5%) during a period of 2 min. Water, almond, and banana extracts were presented twice and amines were presented three times; all odors were freshly prepared for each trial. *Taar2-9*^−/−^ mice and their WT littermates spent similar amounts of time sniffing water (Mann–Whitney *U* test, *U* = 16, *N*_WT_ = 5, *N_Taar2-9_*^-/-^ = 9, *P* = 0.44), almond (*U* = 12, *N_Taar2-9_*^-/-^ = 9, *N*_WT_ = 5, *P* = 0.19), banana (*U* = 18.5, *N_Taar2-9_*^-/-^ = 9, *N*_WT_ = 5, *P* = 0.64), and PEA (*N_Taar2-9_*^-/-^ = 9, *N*_WT_ = 5), but *Taar2-9*^−/−^ mice spent less time sniffing TMA than WT mice (*N_Taar2-9_*^-/-^ = 9, *N*_WT_ = 5). ^∗∗^*P* < 0.05.

*Taar2-9*^−/−^ males and their WT littermates displayed similar and adequate habituation and dishabituation to mouse and non-mouse odors (**Figure 2**). The two types of males also spent similar amounts of time sniffing most non-mouse odors, including β-PEA (**Figure 2**). As expected from the concomitant deletion of TMA-activated TAAR5 (Li et al., 2013), *Taar2-9*^−/−^ males spent less time sniffing TMA than their WT littermates (**Figure 2B**). Thus, *Taar2-9*^−/−^ males had no general deficit in smelling non-mouse odors and the aversive behavior previously reported to be induced by β-PEA-activated TAAR4 (Ferrero et al., 2011; Dewan et al., 2013) was similar for both genotypes even though the *Taar2-9*^−/−^ mice lacked TAAR4, potentially due to lack of sensitivity of our WT mouse strain (C57BL/6) or the experimental setup to recognize β-PEA.

**FIGURE 3:**
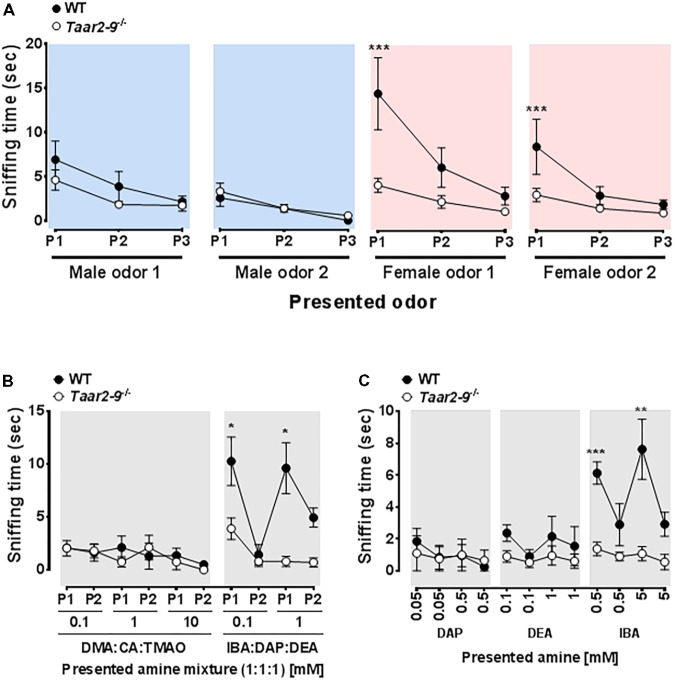
Behavioral response of *Taar2-9*^−/−^ and wild type (WT) males to different odors. Mean (±SEM) periods of time *Taar2-9*^−/−^ and WT males spent sniffing **(A)** male (blue) and female (red) odors, **(B)** mixtures of three amines in different concentrations, and **(C)** single amines in different concentrations during a period of 2 min. Male and female odors were obtained from two different cages inhabited by males (male odor 1 and 2) and females (female odor 1 and 2), respectively. Male and female odors were presented three times (P1–P3) and amine mixtures were presented twice (P1–P2); odors were freshly prepared for each trial. DMA, dimethylamine; CA, cinnamide; TMAO, trimethylamine oxide; IBA, isobutylamine; DAP, diaminopropane; DEA, diethylamine. *N* = 5–9 male mice/group. ^∗^*P* < 0.05, ^∗∗^*P* < 0.01, ^∗∗∗^*P* < 0.001.

Sniffing behavior of the two types of males was also similar when odors from non-familiar WT males were presented (Mann–Whitney *U* tests, male odor 1: *U* = 35, *N_Taar2-9_-*/- = 9, *N*_WT_ = 5, *P* = 0.112; male odor 2: *U* = 21, *N_Taar2-9_-*/- = 9, *N*_WT_ = 5, *P* = 0.90; **Figure 3A**, blue panels). In contrast, when a mixture of odors from females was presented, *Taar2-9*^−/−^ males spent less time sniffing the odor than WT littermates (female odor 1: *U* = 44, *N_Taar2-9_-*/- = 9, *N*_WT_ = 5, *P* = 0.002; female odor 2: *U* = 45, *N_Taar2-9_-*/- = 9, *N*_WT_ = 5, *P* < 0.001; **Figure 3A**, red panels).

### Behavioral Response of *Taar2-9*^-/-^ and WT Males to Exposure to Urinary Amines

Exposure to an odor mixture containing DMA, CA, and TMAO did not induce a difference in sniffing duration between *Taar2-9*^−/−^ and WT males (0.1 mM: *U* = 7, *N_Taar2-9_-*/- = 4, *N*_WT_ = 4, *P* = 0.88; 1 mM: *U* = 11, *N_Taar2-9_-*/- = 4, *N*_WT_ = 4, *P* = 0.46; 10 mM: *U* = 11, *N_Taar2-9_-*/- = 4, *N*_WT_ = 4, *P* = 0.44; **Figure 3B**, left panel). In contrast, when exposed to a mixture containing IBA, DAP, and DEA, *Taar2-9*^−/−^ males spent less time sniffing the more concentrated odor than WT males (0.1 mM: *U* = 16, *N_Taar2-9_-*/- = 4, *N*_WT_ = 4, *P* = 0.081; 1 mM: *U* = 16, *N_Taar2-9_-*/- = 4, *N*_WT_ = 4, *P* = 0.029; **Figure 3B**, right panel).

When presenting IBA alone, *Taar2-9*^−/−^ males spent less time sniffing than WT males for both concentrations (0.5 mM: *U* = 16, *N_Taar2-9_-*/- = 9, *N*_WT_ = 5, *P* = 0.029; 5 mM: *U* = 16, *N_Taar2-9_-*/- = 9, *N*_WT_ = 5, *P* = 0.029; **Figure 3C**). In contrast, DAP and DEA both did not induce a difference in sniffing duration (DAP: 0.05 mM: *U* = 12, *N_Taar2-9_-*/- = 9, *N*_WT_ = 5, *P* = 0.30; 0.5 mM: *U* = 9, *N_Taar2-9_-*/- = 9, *N*_WT_ = 5, *P* = 0.87; DEA: 0.1 mM: *U* = 13.5, *N_Taar2-9_-*/- = 9, *N*_WT_ = 5, *P* = 0.15; 1 mM: *U* = 10, *N_Taar2-9_-*/- = 9, *N*_WT_ = 5, *P* = 0.66; **Figure 3C**).

### Concentration of Odorants in Urine of Female and Male Mice and in Urine of Females in Different Reproductive States

**FIGURE 4:**
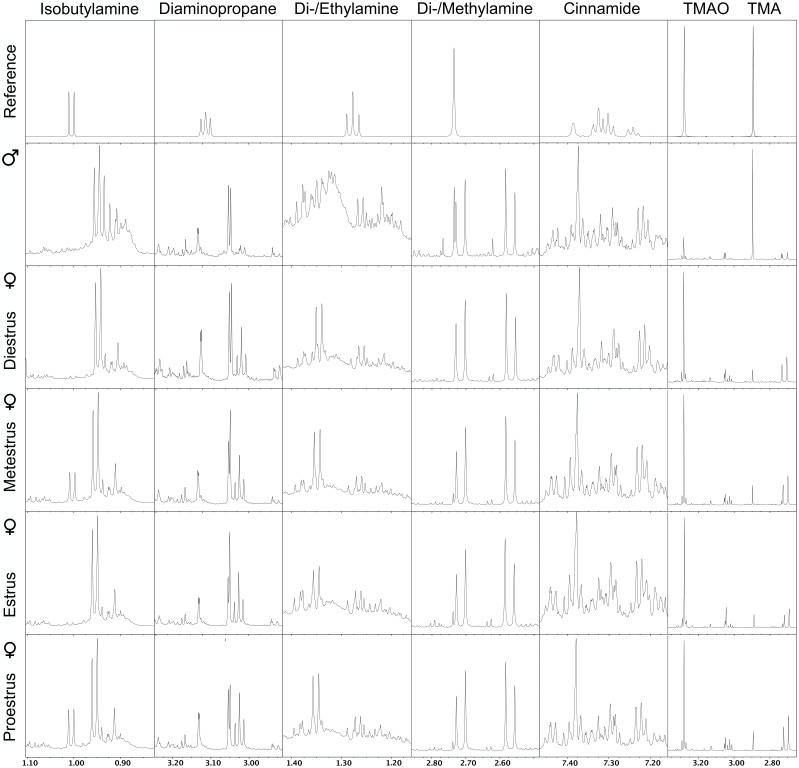
Nuclear magnetic resonance (NMR) spectrograms of urine from wild type male mice and wild type female mice at different stages of the estrus cycle. Shown is one representative spectrogram per sex and estrus cycle stage and for amines with spectra that differed between males and females either by NMR or by GC/MS analysis. TMAO, trimethylamine oxide; TMA, trimethylamine.

**FIGURE 5:**
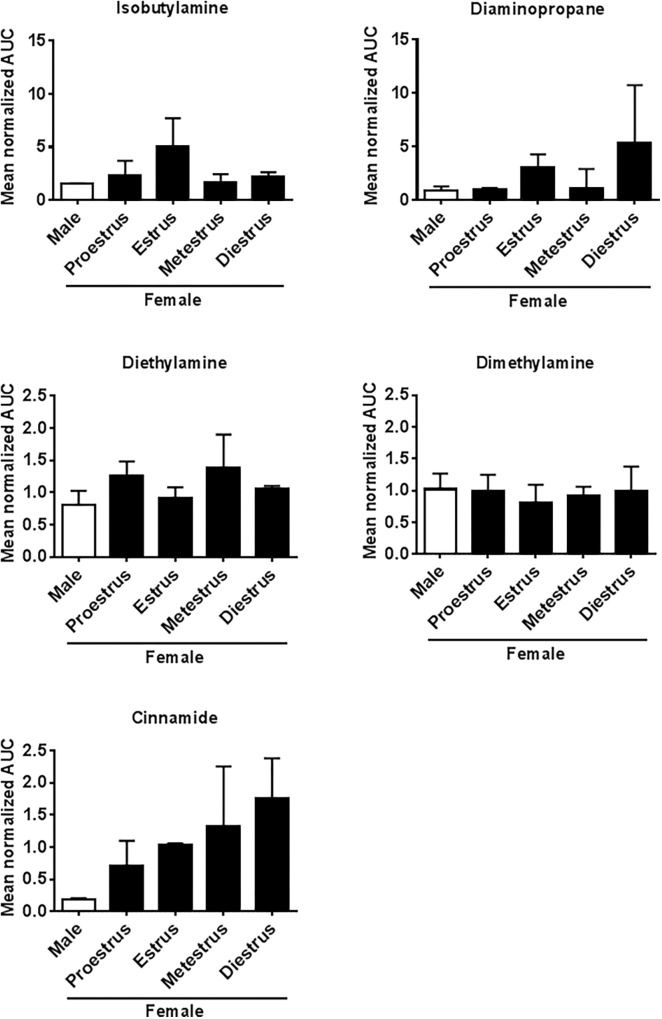
Amine concentrations in urine of male and female mice at different estrus cycle stages. Mean (±SEM) creatinine-normalized AUC of five amines measured using GC-MS.

The NMR spectra of urine of age-matched female and male WT mice differed for the amines IBA, DEA, DMA, CA, TMAO, and TMA (**Figure 4**). Quantitative GC-MS analysis of the average creatinine-normalized AUC of all these amines and DAP revealed that female urine contained higher concentrations of DEA and CA than male urine (Mann–Whitney *U* exact tests; DEA: *U* = 22, *N*_female_ = 8, *N*_male_ = 3, *P* = 0.042; CA: *U* = 18, *N*_female_ = 9, *N*_male_ = 2, *P* = 0.036; **Figure 5**). Female and male urine had similar concentrations of IBA (*U* = 24.5, *N*_female_ = 10, *N*_male_ = 3, *P* = 0.13), DAP (*U* = 18, *N*_female_ = 9, *N*_male_ = 3, *P* = 0.48), and DMA (*U* = 12, *N*_female_ = 12, *N*_male_ = 3, *P* = 0.42). TMAO was not detected and TMA did not derivatize. The average creatinine-normalized AUC of the five tested amines in female urine did not significantly vary with estrus stage (Friedman tests: IBA: Chi^2^ = 5.4, *N* = 2 females, *df* = 3, *P* = 0.14; DEA: Chi^2^ = 1.0, *N* = 2, *df* = 2, *P* = 0.61; DMA: Chi^2^ = 1.0, *N* = 3, *df* = 3, *P* = 0.80; **Figure 5**). Tests for DAP and CA were not performed due to small sample sizes.

### Activation of TAARs by IBA and β-PEA

**FIGURE 6:**
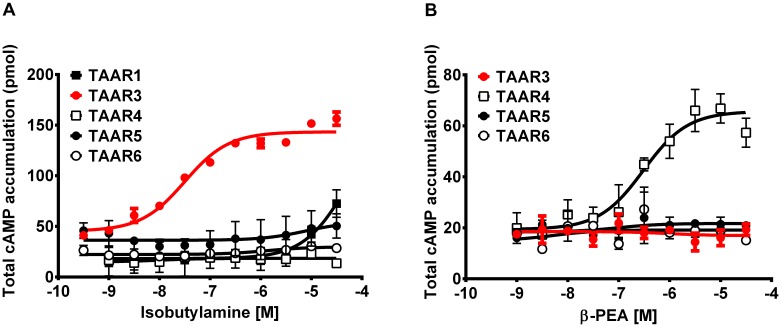
Concentrations of cAMP in TAAR-expressing cells stimulated with two amines. Mean (±SEM) concentrations of cAMP in HEK 293 cells expressing TAAR1, TAAR3 and Gαolf, TAAR4, TAAR5, and TAAR6 when stimulated with different concentrations of **(A)** isobutylamine and **(B)** β-phenylethylamine (β-PEA).

Cells expressing TAAR3 accumulated significantly higher concentrations of cAMP when stimulated with IBA (EC_50_ = 32 nM) than cells expressing TAAR1, TAAR4, TAAR5 and TAAR6 (**Figure 6A**). In contrast, stimulation by β-PEA, a known agonist of TAAR1 and TAAR4, did not result in stimulation of cells expressing TAAR3 (**Figure 6B**). As expected, β-PEA induced accumulation of more cAMP in TAAR4-expressing cells (**Figure 6B**).

### Effect of Olfactory Stimulation With IBA and Trimethylthiazoline (TMT) on Neural Activity of *Taar2-9*^-/-^ and WT Mice

**FIGURE 7:**
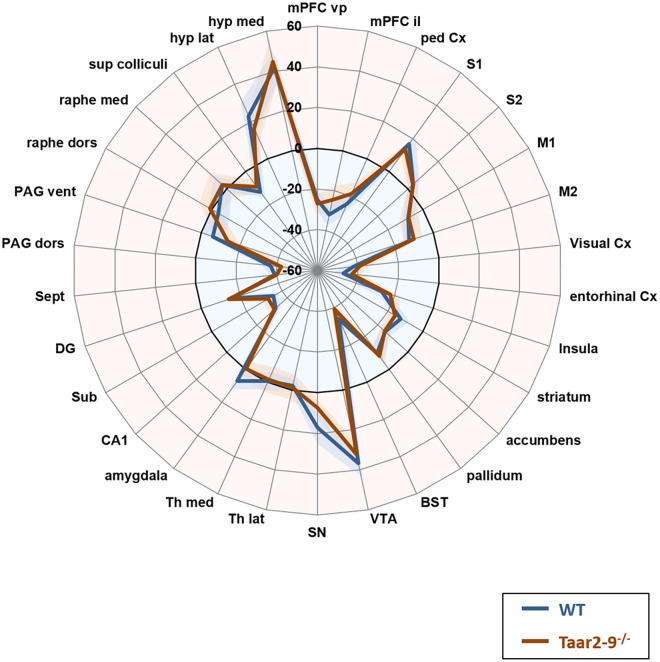
Neural activity in odor-naïve *Taar2-9*^−/−^ and wild type (WT) littermate mice as assessed by fMRI. Neural activity of odor-naïve *Taar2-9*^−/−^ and WT littermate mice was assessed by continuous arterial spin labeling functional magnetic resonance imaging (CASL-fMRI) which reports cerebral perfusion as a proxy for neural activity. Data are means ± SEM (solid lines and shaded area, respectively); *N* = 11 to 12 per group; differences were tested region-wise for significance using two-way ANOVA. mPFC vp, ventral prelimbic medial prefrontal cortex; mPFC il, ventral infralimbic medial prefrontal cortex; ped Cx, dorsal peduncular cortex; S1, primary somatosensory cortex; S2, secondary somatosensory cortex; M1, primary motor cortex; M2, secondary motor cortex; Cx, cortex; BST, bed nucleus of the stria terminalis; VTA, ventral tegmental area; SN, substantia nigra; Th lat, lateral thalamus; Th med, Medial thalamus; CA1, hippocampal area CA1; Sub, subiculum; DG, dentate gyrus; Sept, septum; PAG dors, dorsal periaqueductal gray; PAG vent, ventral periaqueductal gray; raphe dors, dorsal raphe; raphe med, median raphe; sup colliculi, superior colliculus; hyp lat, lateral hypothalamus; hyp med, medial hypothalamus.

**FIGURE 8:**
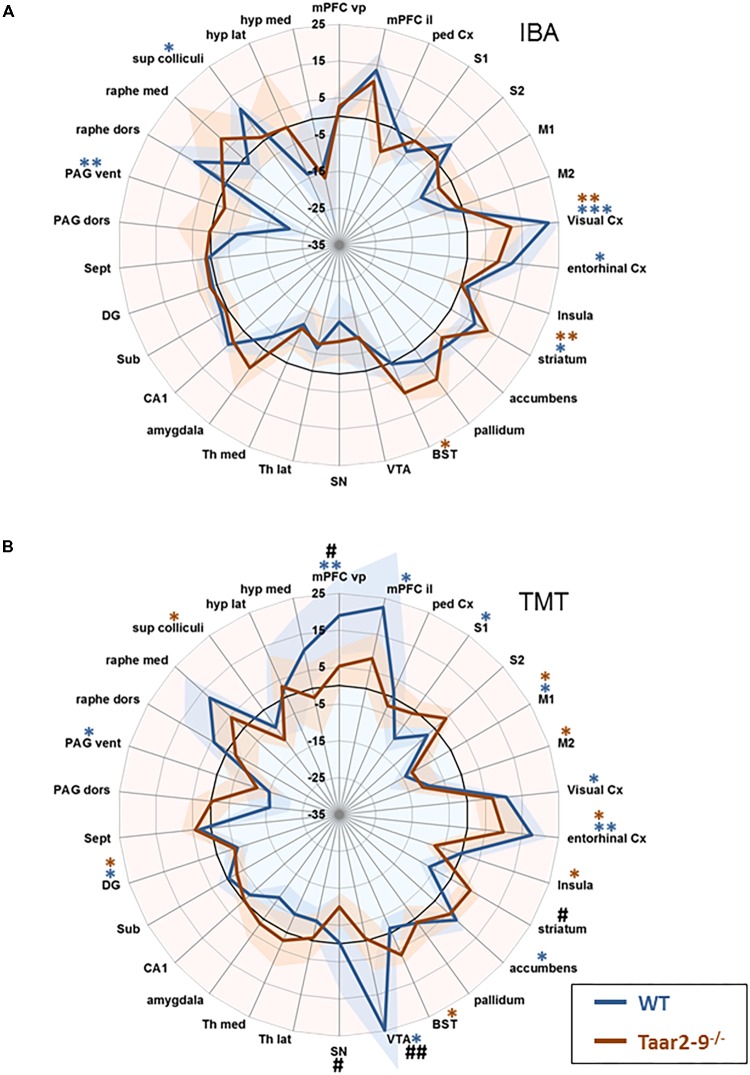
Region-specific neural activity of *Taar2-9*^−/−^ and wild type (WT) mice stimulated with isobutylamine (IBA) and trimethylthiazoline (TMT). Neural activity was assessed by functional magnetic resonance imaging (fMRI) based on continuous arterial spin labeling (CASL) which reports cerebral perfusion as a proxy for neural activity. Represented are means (solid lines) ± SEM (shaded area) of region-specific normalized perfusions elicited by **(A)** IBA and **(B)** TMT of 7 to 12 *Taar2-9*^−/−^ males and WT littermates. Blue ^∗^ and red ^∗^ denote statistically significant (^∗^*P* < 0.05, ^∗∗^*P* < 0.01, ^∗∗∗^*P* < 0.001) differences between odor exposed and non-exposed *Taar2-9*^−/−^ and WT mice, respectively. ^#^Denotes statistically significant (^#^*P* < 0.05, ^##^*P* < 0.01) differences between *Taar2-9*^−/−^ and WT mice. mPFC vp, ventral prelimbic medial prefrontal cortex; mPFC il, ventral infralimbic medial prefrontal cortex; ped Cx, dorsal peduncular cortex; S1, primary somatosensory cortex; S2, secondary somatosensory cortex; M1, primary motor cortex; M2, secondary motor cortex; Cx, cortex; BST, bed nucleus of the stria terminalis; VTA, ventral tegmental area; SN, substantia nigra; Th lat, lateral thalamus; Th med, Medial thalamus; CA1, hippocampal area CA1; Sub, subiculum; DG, dentate gyrus; Sept, septum; PAG dors, dorsal periaqueductal gray; PAG vent, ventral periaqueductal gray; raphe dors, dorsal raphe; raphe med, median raphe; sup colliculi, superior colliculus; hyp lat, lateral hypothalamus; hyp med, medial hypothalamus.

Baseline comparison of naïve males, i.e., *Taar2-9*^−/−^ and WT mice that had not been previously exposed to the odors of interest, showed no significant difference in their neural activity patterns (**Figure 7**). When awake, naïve mice were presented with IBA for 20 min prior to fMRI examination WT mice had increased neural activity in the visual and entorhinal cortices as well as in superior colliculus. Moreover, changes were seen in brain regions responsible for movement planning and initiation, (increased activity in the striatum) and the coordination of autonomic behavioral response (decreased activity in the ventral periaqueductal gray) (**Figure 8A** and **Supplementary Table S1**). *Taar2-9*^−/−^ mice showed increased activity in the visual cortex, striatum and bed nucleus of the stria terminalis (**Figure 8A** and **Supplementary Table S1**).

When males were stimulated with TMT, an odorant of fox feces that induces unconditioned fear and defensive behaviors in rodents (Kessler et al., 2012), neural activity of *Taar2-9*^−/−^ and WT mice differed from baseline activity in a number of regions. In WT males, the brain regions responsible for cortical assessment (ventral and infralimbic medial prefrontal cortex), emotion-cognition integration (entorhinal cortex), and emotion-motivation integration (ventral tegmental area) were particularly active (**Figure 8B** and **Supplementary Table S1**). WT mice also had increased activity in the visual cortex and accumbens, and decreased activity in the primary somatosensory cortex, primary motor cortex, dentate gyrus and ventral periaqueductal gray (**Figure 8B** and **Supplementary Table S1**). In *Taar2-9*^−/−^ mice, the primary motor cortex, entorhinal cortex, bed nucleus of the stria terminalis, were particularly active whereas activity in the secondary motor cortex, insula, dentate gyrus and the superior colliculus was reduced (**Figure 8B** and **Supplementary Table S1**).

*Taar2-9*^−/−^ and WT mice showed specific differences in their neural activity pattern (**Figure 8B** and **Supplementary Table S1**). One of the brain regions responsible for cortical assessment (i.e., the ventral medial prefrontal cortex), the region responsible for emotion-motivation integration (i.e., the ventral tegmental area), and the substantia nigra were all less active in *Taar2-9*^−/−^ than in WT mice. In contrast, striatal activity was higher in *Taar2-9*^−/−^ than in WT mice (**Supplementary Table S1**).

### Quality and Type of Ultrasonic Vocalizations by *Taar2-9^-/-^* Males and Their WT Littermates

**FIGURE 9:**
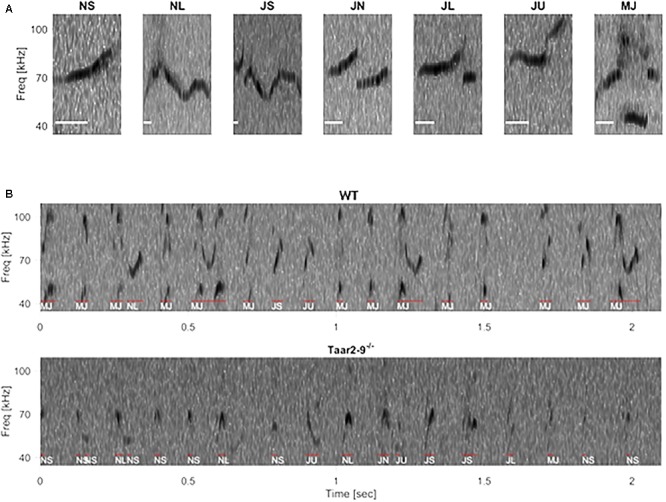
Example spectrogram of ultrasonic vocalizations (USVs) in wild type (WT) and *Taar2-9*^−/−^ littermates. **(A)** Frequency spectrogram of seven typical USV types: NS (call duration ≤ 25 ms), NL (call duration ≥ 25 ms), JS (downward jump > 4 kHz, ratio < 0.4), JN (downward jump > 4 kHz, 0.4 ≤ ratio ≤ 2), JL (downward jump > 4 kHz, ratio > 2), JU (upward jump > 7 kHz), MJ (more than one jump), scale bar: 10 ms. **(B)** Frequency spectrograms of a 2-s period USV of a male WT (Upper) and a male *Taar2-9*^−/−^ (Lower) mouse. Individual syllables, as identified and classified by an automated algorithm, are indicated by red lines and white texts below.

**FIGURE 10:**
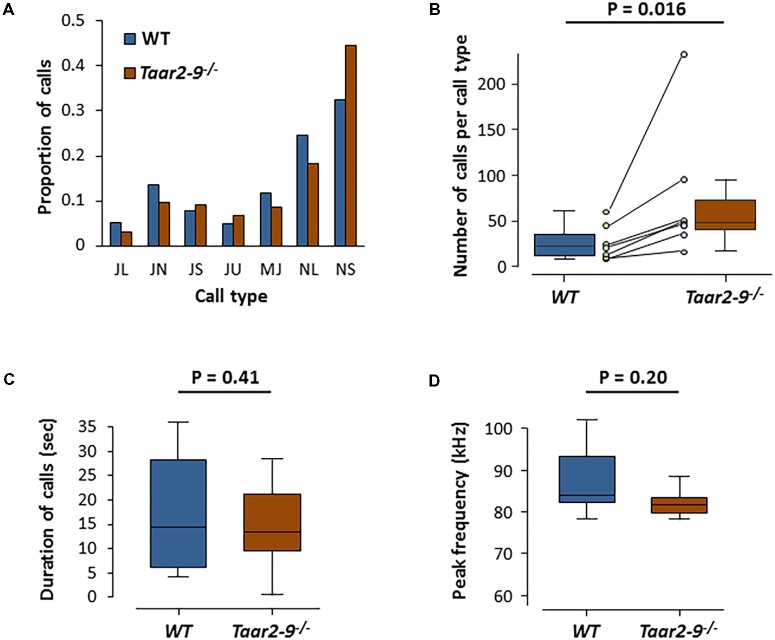
Ultrasonic vocalization (USV) by *Taar2-9*^−/−^ and wild type (WT) male mice when exposed to urine of a female in estrus. **(A)** Percentage of ultrasonic calls of the seven types emitted by *Taar2-9*^−/−^ males (*N* = 7) and WT males (*N* = 4). **(B)** Number of calls per call type. Dots represent the mean numbers of the seven call types emitted by *Taar2-9*^−/−^ (violet) and WT (green) males. **(C)** Cumulative duration of calls. **(D)** Peak frequency of calls emitted during a 5 min session. JL: downward jump >4 kHz, ratio > 2; JN: downward jump >4 kHz, 0.4 ≤ ratio ≤ 2; JS: downward jump >4 kHz, ratio < 0.4; JU: upward jump >7 kHz; MJ: multiple jumps; NL: call duration ≥25 ms; NS: call duration ≤25 ms. Boxes indicate the interquartile range around the median (horizontal bar) and vertical bars represent numbers, durations and peak frequencies that lie within 1.5 times the interquartile range.

When exposed to the urine of a female in estrus, *Taar2-9*^−/−^ and their WT littermates emitted the same types of ultrasonic calls (**Figures 9A,B**). The two types of males did not differ in the distribution of the number of calls emitted across the seven call types (Zero-inflated generalized linear mixed-effects model, *N_Taar2-9_^-^*^/^*^-^* = 7, *N*_WT_ = 4, model estimate = -21.03, *Z* = -0.004, *P* = 0.997; **Figure 10A**). *Taar2-9*^−/−^ males emitted more calls per call type (74.9 ± 28.0) than WT males (26.8 ± 7.4; Wilcoxon signed-rank text, *V* = 28, *N* = 7 call types, *P* = 0.016; **Figure 10B**). The cumulative duration of the different calls was similar for *Taar2-9*^−/−^ and WT males (*U* = 19, *N_Taar2-9_^-^*^/^*^-^* = 7, *N*_WT_ = 4, *P* = 0.41; **Figure 10C**) and also the peak frequency of calls emitted by the two types of males was similar (*U* = 9, *N_Taar2-9_^-^*^/^*^-^* = 7, *N*_WT_ = 4, *P* = 0.20; **Figure 10D**).

### Pro-social and Avoidance Behavior by *Taar2-9^-/-^* Males and Their WT Littermates

**FIGURE 11:**
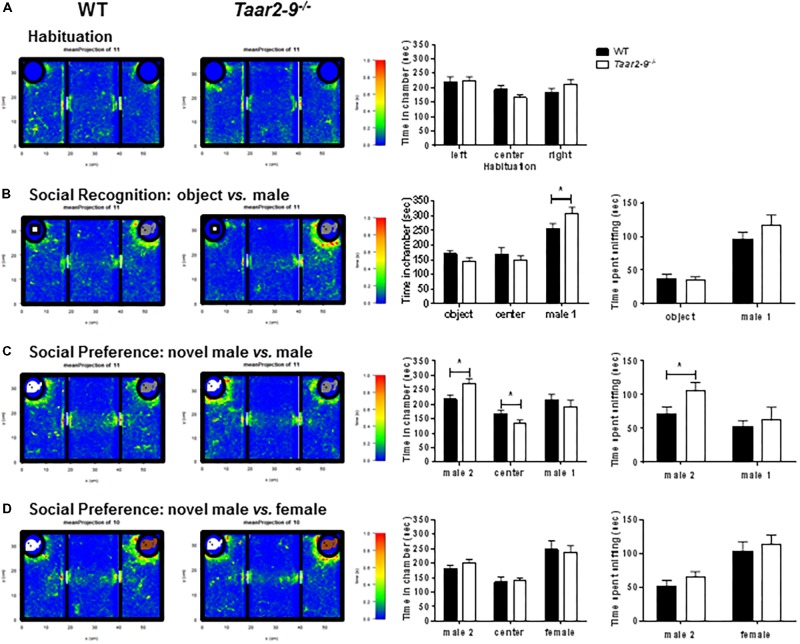
Attraction of *Taar2-9*^−/−^ and wild type (WT) male mice to objects and familiar and unfamiliar conspecifics. Mean (±SEM) periods of time 10–14 focal male mice spent **(A)** in each of three empty chambers after 10 min habituation in the central arena; **(B)** in the three chambers when a novel object (Left) and an unfamiliar male mouse (Right, male 1) were placed into the polycarbonate wire enclosure, and the time they spent sniffing the object and the male, respectively; **(C)** in the three chambers when, after 10 min, the object was replaced by a male mouse (Left, male 2), and the time spent sniffing the two males; **(D)** in the three chambers when male 1 was replaced by a female mouse (Right), and the time spent sniffing the male and the female, respectively. There is no significant difference between WT and *Taar2-9*^−/−^ mice in the recognition and interaction of female mice. ^∗^*P* < 0.01 for the comparison between genotypes; differences were tested using two-way ANOVA followed by Bonferroni’s *post hoc* tests.

*Taar2-9*^−/−^ and WT mice did not express a preference for a specific side in a three-chamber test with empty chambers (**Figure 11A**). However, *Taar2-9*^−/−^ mice spent more time in the chamber with a mouse (male 1) compared to WT littermates (**Figure 11B**). Furthermore, *Taar2-9*^−/−^ mice spent less time in the chamber with a novel male mouse (male 2), more time in the center, and, importantly, more time sniffing the novel male mouse (male 2), indicating an increase in social recognition (**Figure 11C**). Presenting a female mouse instead of a male mouse revealed that both male genotypes spent more time in the chamber with a novel male (male 2) than the familiar female, but less time sniffing the novel male than the familiar female (**Figure 11D**). Thus, the presence of a female mouse with the combination of odorant and non-odorant social cues represents a strong signal which is not altered by deleting the TAAR2-9 cluster of olfactory receptors.

**FIGURE 12:**
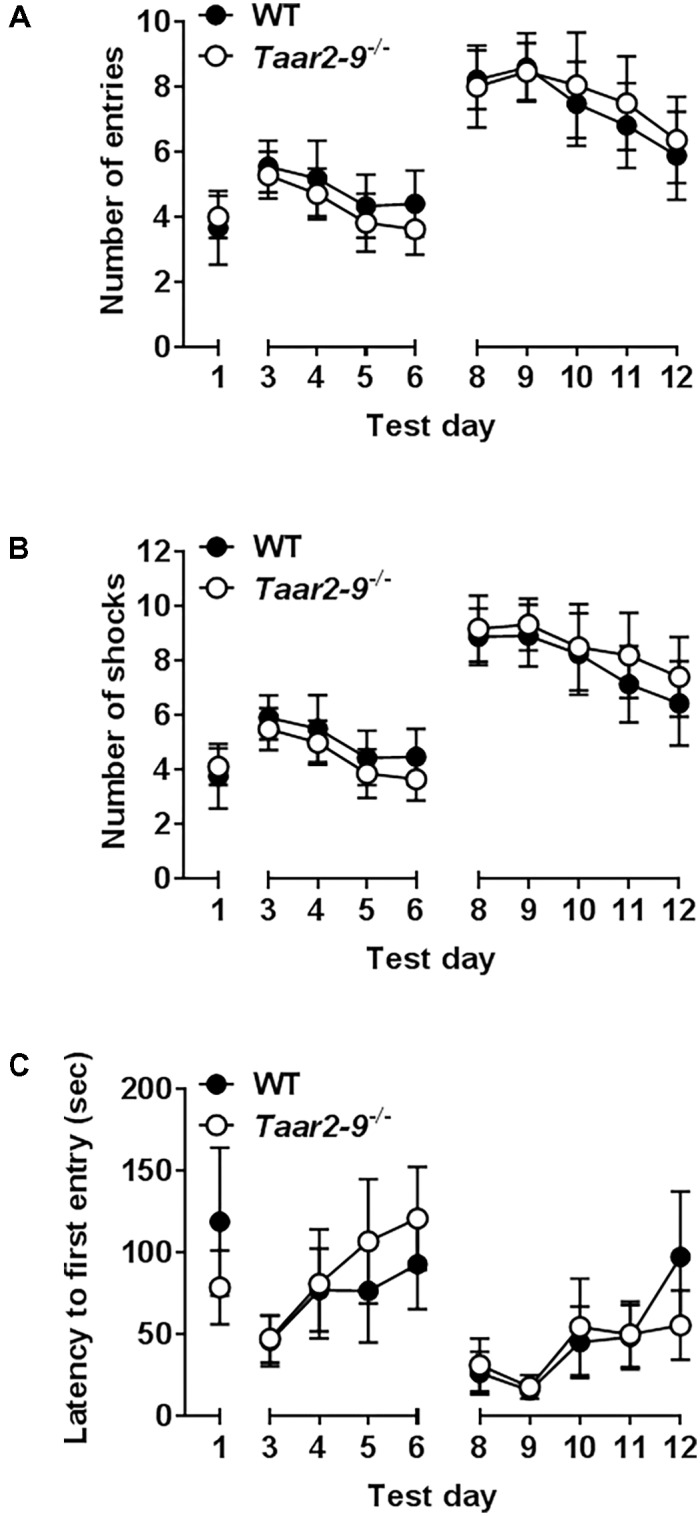
Responses of *Taar2-9*^−/−^ male mice and wild type (WT) littermates in active place avoidance test. First 4 days in the place avoidance training apparatus represent the acquisition of the memory necessary to perform the test. From day six on the shock zone is reversed and entries into the shock zone **(A)**, shocks per day **(B)** and latency to first entry into the shock zone **(C)** are measured. Both WT and *Taar2-9*^−/−^ mice (*N* = 8–9 per group) showed the same memory formation and cognitive flexibility. Thus neither hippocampus nor amygdala, which are responsible for the associative learning seem to be impaired in *Taar2-9*^−/−^ mice. Data are means ± SEM; two-way ANOVA comparing WT with *Taar2-9*^−/−^ animals.

*Taar2-9*^−/−^ mice and their WT littermates did not differ in any paradigm of the active avoidance task (**Figures 12A–C**).

### AMPH-Induced Locomotor Activity of *Taar2-9^-/-^* and Their WT Littermates

**FIGURE 13:**
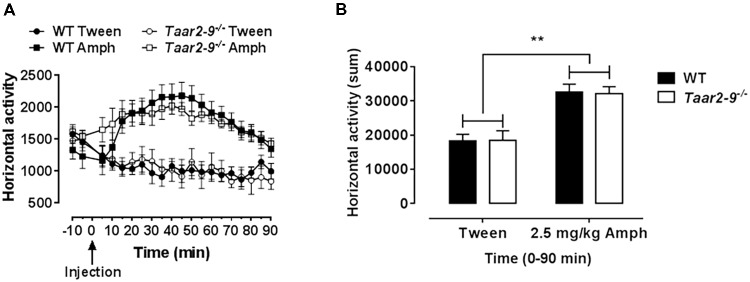
Drug-induced hyperactivity in *Taar2-9*^−/−^ mice is not altered. **(A)** Locomotor activity of *Taar2-9*^−/−^ and their wild type (WT) littermates was measured after application of d-AMPH (2.5 mg/kg i.p.) or vehicle (Tween). **(B)** Cumulated locomotor activity for the 90 min following AMPH treatment. No altered sensitivity was detected comparing the strains. *N* = 10–14 per group. Data represent mean ± SEM; ^∗∗^*P* < 0.01, one-way ANOVA followed by Tukey’s *post hoc* test.

Horizontal activity of mice prior to AMPH challenge showed no difference between *Taar2-9*^−/−^ and WT littermates. While AMPH challenge induced an increased locomotor activity in both mouse lines due to dopamine release, no difference was detected between *Taar2-9*^−/−^ and WT mice (**Figures 13A,B**).

## Discussion

Our study revealed a transduction pathway that links a volatile amine (IBA) contained in the urine of female mice to behaviors in males that are indicative of sexual interest. We demonstrate that IBA activates a specific receptor (TAAR3) that is expressed by neurons of the MOE and that activates brain regions associated with sexual behavior. We also show that male mice lacking TAARs are less attracted to females than WT males. This is the first demonstration of a comprehensive transduction pathway linking odorants to TAARs and male behavior related to the recognition of – and sexual interest in – females. It is also the first time that an odorant was shown to be linked to such behavior without the direct involvement of components of the VNO. These results may be relevant for humans because humans lack a VNO but do have TAARs in the MOE like mice and many other mammals (Frasnelli et al., 2011; Matsuo et al., 2015).

Our measurements of cAMP concentrations in TAAR-expressing cells stimulated with different amines demonstrated that IBA selectively and potently activates TAAR3. We also found that *Taar2-9*^−/−^ male mice (that lack TAAR3) spent less time sniffing IBA than WT males, confirming the tight association between IBA and TAAR3. TAAR3 was previously reported to be activated *in vitro* by another volatile amine, isoamylamine (Liberles and Buck, 2006), which has been described as a male-derived pheromone able to accelerate puberty onset in female mice (Nishimura et al., 1989). This suggests that TAAR3 plays an important role for the detection of social cues influencing the reproductive behavior and physiology in mice. In our study, *Taar2-9*^−/−^ males spent less time sniffing female than male odors, suggesting that TAAR3 is also important for the discrimination between female and male odors and/or the attraction to female odors. Previous studies have described several pheromones that help individuals recognize conspecifics and potential mating partners (Keverne, 1999; Del Punta et al., 2002; Baum and Kelliher, 2009). Our results suggest that IBA may be an additional cue for males to recognize females and an important odorant for males to be attracted to females.

Interestingly, a recent study detected a significant interaction between mate choice and the female TAAR3 genotype, with TAAR3-heterozygous females being more likely to choose major histocompatibility complex-diverse males (Santos et al., 2016). Their results suggest that TAAR3 and olfactory cues are key mediators in mammalian major histocompatibility complex-dependent mate choice. Our results on the activation of TAAR3-expressing cells by the volatile amine IBA present in female urine, the activation of brain regions associated with sexual behavior and the expression of sexual interest by males when exposed to female urine (but not when exposed to male urine) suggests that TAAR3 and IBA are involved in the communication between potential mates.

Isobutylamine was the only amine found to differ between male and female urine and that at the same time resulted in a different sniffing behavior when presented to *Taar2-9*^−/−^ and WT mice due to its activation of the TAAR family member TAAR3. We do not know the relevance of the other odors DMA, CA, TMAO, DAP, and DEA, but they likely do not act via TAAR2-9 and bind to a different set of receptors. It will be interesting to explore the function of these amines in future studies.

We found that WT and *Taar2-9*^−/−^ mice showed differences in the activity of various brain regions when exposed to IBA compared to baseline activities. WT mice had higher neural activity in the visual and entorhinal cortices, superior colliculus, and the brain regions responsible for movement planning and initiation, while lower neural activity in brain areas responsible for the coordination of autonomic behavioral response. Upon exposure to IBA, *Taar2-9*^−/−^ mice showed increased activity in the visual cortex, striatum and bed nucleus of the stria terminalis. These brain regions are associated with sexual behaviors, suggesting that the presence of TAAR2-9 is important for the activation of sexual interest of males and other behaviors associated with mate choice. The combination of activation of various additional single brain regions that were, individually, statistically insignificant indicate additional interesting and meaningful differences in brain activity between *Taar2-9*^−/−^ and WT mice. The increased activity in the bed nucleus of the stria terminalis, a tendency toward increased neural activity in the ventral pallidum, amygdala and the median raphe, combined with a tendency toward a reduced activity in the ventral PAG and medial hypothalamus of *Taar2-9*^−/−^ mice are consistent with previous reports in which a similar set of brain regions was shown to be activated upon exposure to cues inducing rodent mating behavior (Dulac and Wagner, 2006; Hashikawa et al., 2016).

In rodents, exposure to TMT, a non-amine like odorant present in fox urine, induces a very strong fear response. Even though TMT is not known to bind to any TAAR receptor, the stimulation of *Taar2-9*^−/−^ mice resulted in an altered brain activation pattern (decreased activity in the ventral tegmental area and medial prefrontal cortex), which can be interpreted as a faster recovery after the exposure to a stimuli inducing unconditioned fear. Exposure to TMT caused aberrant activity in brain regions involved in the regulation of aversive emotional arousal (ventral tegmental area) and movement planning and initiation (striatum). These results were similar to those previously reported for WT rats (Kessler et al., 2012) and confirmed a possible role for TAARs in the regulation of innate fear.

The presence of TAAR2-9 did not seem to affect the courtship behavior of males when they were exposed to the odor of females in estrus. The types, duration and frequency of calls emitted by *Taar2-9*^−/−^ males and WT males were similar to those emitted by laboratory (Whitney and Nyby, 1979) and wild (Musolf et al., 2010) male mice when they court females and during mating. The only difference was that *Taar2-9*^−/−^ males emitted more calls per call type than WT males.

The concentration of IBA in the urine of female mice varied across the estrus cycle and concentration during estrus was higher than before and after estrus in all three females measured. Being able to detect when a female is in estrus is important for males, particularly in polygynous species, because it allows them to focus their courtship investment on those females that are most likely to produce offspring with them (Nunn, 1999). Confirmation of our results in a few additional females would thus provide further evidence that IBA and TAARs play an important role in the recognition of breeding partners and mate choice. The physiological mechanisms underlying changes in IBA concentration remain to be determined. Endogenously, IBA is generated from L-valine by the valine decarboxylase, and is rapidly transformed into isobutylhydroxylamine by the IBA N-hydroxylase (Kyoto Encyclopedia of Genes and Genomes^1^). IBA is a volatile amine with a short half-life. This may explain the relatively low concentration of IBA in the urine of female mice. It also suggests that the use of this cue may be restricted in time and the behavioral phenotype induced in male mice expressed only during a short period of time. In accordance with this idea, previous studies on wild house mice found that soiled female urine and urine exposed to air elicited fewer USVs from males than fresh female urine, which might be caused by loss of volatiles in bedding (Nyby and Zakeski, 1980; Musolf et al., 2010). It also matches the observation in free-ranging animals of various taxa that males try to sniff the females’ urine as shortly after the female urinated as possible (Hart, 1983).

The deletion of TAAR2-9 induced changes in the interest of males in females but it did not induce significant changes in other behaviors. *Taar2-9*^−/−^ mice and their WT littermates did not differ from each other in terms of memory and learning performance in the active place avoidance test. This suggests no or only a minor impact of TAAR2-9 on the hippocampus and amygdala, the brain regions that have substantial neuroplastic potential and are known to play an important role in various cognitive functions as well as effective information processing (Bannerman et al., 2004; Davidson and Jarrard, 2004). *Taar2-9*^−/−^ and WT mice also did not differ in the assessment of associative learning and memory. Furthermore, *Taar2-9*^−/−^ mice expressed normal locomotor activity, co-ordination and balance, indicating functional integrity of the brainstem and cerebellum. This is in contrast to *Taar1^-^*^/^*^-^* mice who modulate monoaminergic neurotransmission in brain regions containing dopaminergic and serotonergic neurons like the ventral tegmental area (Llinas and Welsh, 1993; Lalonde and Strazielle, 2007). *Taar2-9*^−/−^ and WT littermates also formed the same call types, suggesting that there is no difference in the ability of the two types of males to vocalize. Finally, *Taar2-9*^−/−^ mice and their WT littermates showed similar avoidance behavior, indicating that the ability of *Taar2-9*^−/−^ mice to avoid an aversive event by learning to perform a specific behavior in response to a stimulus cue is not altered.

## Conclusion

We here describe for the first time a comprehensive transduction pathway that links odorants with TAARs and male sexual interest in females. We show that a volatile amine contained in the urine of female mice, IBA, is a potent ligand for TAAR3 that activates brain regions associated with sexual behaviors of males. We also show that males lacking TAAR3 and other TAARs do not recognize or show interest in females. Our results further suggest that variation in IBA may represent a simple olfactory cue for males to recognize receptive females. Our results are consistent with the hypothesis that IBA and TAARs play an important role in the recognition of breeding partners and mate choice. Knowing the mechanism by which males acquire information about potential mates and the components that are involved in the induction of male sexual interest helps us understand how male mammals recognize females and how they know when it is best for them to court a female. This may help us understand better which males decide to court and mate with females and how the sexual interest of males can be stimulated.

## Author Contributions

AH, CM, AS, FC, MP, BK, AI, and MH designed the studies. AH, CM, AS, FC, MP, Y-PZ, BK, and MH conducted the experiments and analyzed the data. AH, OH, and MH wrote the manuscript.

## Conflict of Interest Statement

All authors with the exception of MP and OH are employees of F. Hoffmann-La Roche Ltd. MP was an employee of F. Hoffmann-La Roche Ltd. and declares no conflict of interest. OH declares no conflict of interest. The handling Editor declared a past co-authorship with one of the authors, MH.

## Footnotes

1 http://www.genome.jp/kegg/pathway.html

## Abbreviations

AMPH: amphetamine
AUC: area under the curve
β-PEA: β-phenylethylamine
CA: cinnamide
DAP: diaminopropane
DEA: diethylamine
DMA: dimethylamine
fMRI: functional magnetic resonance imaging
GC-MS: gas chromatography mass spectrometry
IBA: isobutylamine
MOE: main olfactory epithelium
NMR: nuclear magnetic resonance
OR: odorant receptors
TAAR: trace amine-associated receptor
TMA: trimethylamine
TMAO: trimethylamine oxide
TMT: trimethylthiazoline
USV: ultrasonic vocalization
VNO: vomeronasal organ
WT: wild type
