# Supplementary material for: How Female Mice Attract Males: A Urinary Volatile Amine Activates a Trace Amine-Associated Receptor That Induces Male Sexual Interest
Source: Front Pharmacol. 2018 Aug 15;9:924. doi: 10.3389/fphar.2018.00924 (PMC6104183; doi:10.3389/fphar.2018.00924)
Supplement: Supplementary file 1 [file Table_1.DOCX]

**SUPPLEMENTARY MATERIAL**

**Table S1: Normalized perfusion as proxy for neural activity as assessed by fMRI in *Taar2-9*^-/-^ and wild type (WT) littermates exposed to isobutylamine (IBA) or trimethylthiazoline (TMT).**

Values show difference to odor-naïve controls of the respective genotype. Data represent mean ± SEM for group sizes of 7 to 12 animals. Means of normalized perfusion were tested region-wise for significant differences using two-way ANOVAs with the factors genotype and odor, followed by post-hoc contrasts for genotype differences and genotype-odor interactions. * Denote statistically significant (*P < 0.05, **P < 0.01, ***P < 0.001) differences between odor exposed and non-exposed *Taar2-9^-/-^* and WT mice, respectively. ^#^ Denotes statistically significant (^#^P < 0.05, ^##^P < 0.01) differences between *Taar2-9*^-/-^ and WT mice.

|  | **Normalized perfusion (% difference to respective odor-naïve control)** | | | | | | | |
| --- | --- | --- | --- | --- | --- | --- | --- | --- |
|  | **IBA** | | | | **TMT** | | | |
| **Brain** | **WT** | | ***Taar2-9*^-/-^** | | **WT** | | ***Taar2-9*^-/-^** | |
| **Region** | **Mean** | SEM | **Mean** | SEM | **Mean** | SEM | **Mean** | SEM |
| mPFC vp | 2.1 | 4.0 | 2.8 | 4.8 | 19.1^** #^ | 10.9 | 5.2^#^ | 5.0 |
| mPFC il | 13.5 | 5.8 | 10.4 | 7.7 | 22.4^*^ | 11.5 | 8.3 | 7.6 |
| ped Cx | 1.8 | 5.4 | -7.3 | 5.6 | 1.5 | 7.6 | -2.6 | 7.0 |
| S1 | -3.7 | 4.6 | 0.0 | 4.6 | -9.4^*^ | 4.1 | -1.1 | 3.8 |
| S2 | 5.7 | 4.0 | 0.7 | 3.8 | -2.8 | 3.0 | 4.0 | 3.5 |
| M1 | -9.2 | 4.4 | -3.7 | 6.8 | -14.2^*^ | 6.3 | -12.2^*^ | 5.2 |
| M2 | -3.7 | 4.0 | -1.5 | 7.0 | -9.4 | 4.7 | -11.1^*^ | 4.5 |
| Visual Cx | 22.1^***^ | 4.4 | 11.9^**^ | 4.0 | 10.7^*^ | 3.5 | 7.0 | 4.7 |
| entorhinal Cx | 12.2^*^ | 5.1 | 8.5 | 4.8 | 17.6^**^ | 4.9 | 9.9^*^ | 5.6 |
| Insula | 1.6 | 2.8 | 0.0 | 5.1 | -0.5 | 3.6 | -7.6^*^ | 4.0 |
| striatum | 7.6^*^ | 3.8 | 11.5^**^ | 3.7 | -6.7^#^ | 4.0 | 6.1^#^ | 3.1 |
| accumbens | 5.1 | 3.1 | 2.6 | 2.8 | 7.8^*^ | 2.8 | 5.4 | 3.4 |
| pallidum | 3.8 | 4.1 | 10.0 | 5.7 | 1.2 | 5.1 | 1.0 | 5.2 |
| BST | 0.4 | 4.1 | 8.9^*^ | 2.8 | -1.2 | 3.7 | 6.7^*^ | 2.7 |
| VTA | -8.8 | 10.1 | -9.3 | 8.1 | 24.8^* ##^ | 15.5 | -0.7^##^ | 8.7 |
| SN | -14.1 | 8.6 | -8.9 | 5.8 | -0.2^#^ | 13.2 | -9.9^#^ | 4.4 |
| Th lat | -6.3 | 3.3 | -7.6 | 6.6 | -5.6 | 3.7 | -0.9 | 7.0 |
| Th med | -11.4 | 5.1 | -10.2 | 7.0 | -5.4 | 4.6 | 2.4 | 7.7 |
| amygdala | -4.2 | 5.8 | 6.3 | 7.7 | -7.1 | 5.1 | 1.5 | 5.5 |
| CA1 | 5.4 | 2.0 | 3.8 | 3.2 | -2.5 | 3.1 | -0.3 | 2.6 |
| Sub | 1.9 | 2.3 | 0.5 | 3.0 | -0.4 | 3.5 | -3.0 | 3.1 |
| DG | 1.1 | 2.0 | 1.8 | 3.8 | -6.0^*^ | 3.1 | -5.3^*^ | 2.2 |
| Sept | 1.1 | 2.8 | 1.4 | 2.8 | 3.0 | 2.9 | 4.3 | 3.8 |
| PAG dors | -7.0 | 5.0 | 0.4 | 8.7 | -16.1 | 5.8 | -0.3 | 6.0 |
| PAG vent | -20.8^**^ | 6.6 | -2.4 | 7.9 | -15.1^*^ | 7.0 | -11.6 | 7.3 |
| raphe dors | 10.2 | 9.4 | 1.6 | 7.9 | 4.3 | 8.5 | -2.6 | 7.2 |
| raphe med | -1.9 | 6.2 | 8.1 | 13.0 | 12.2 | 9.0 | 4.4 | 9.5 |
| sup colliculi | 10.6^*^ | 4.9 | 1.2 | 6.2 | -5.7 | 2.4 | -9.8^*^ | 3.9 |
| hyp lat | -13.9 | 10.5 | 0.0 | 16.8 | 2.1 | 12.0 | 3.0 | 9.9 |
| hyp med | -13.8 | 16.3 | -16.4 | 10.4 | 10.6 | 9.4 | -2.6 | 9.9 |
